# Editorial: Women in Hepato Pancreatic Biliary (HPB) Tumors: 2021, Volume I

**DOI:** 10.3389/fonc.2022.927775

**Published:** 2022-06-23

**Authors:** Nadia M. Hamdy, Yuming Jiang, Sumit Sahni, Wenchuan Wu, Yuelun Zhang, Bhawna Sirohi, Jiang Chen

**Affiliations:** ^1^ Department of Biochemistry and Molecular Biology, Faculty of Pharmacy, Ain Shams University, Cairo, Egypt; ^2^ Radiation Oncology Department, Stanford University, Stanford, CA, United States; ^3^ Kolling Institute of Medical Research, Royal North Shore Hospital, Sydney, NSW, Australia; ^4^ Department of General Surgery, Zhongshan Hospital, Fudan University, Shanghai, China; ^5^ Medical Research Center, Peking Union Medical College Hospital, Chinese Academy of Medical Sciences and Peking Union Medical College, Beijing, China; ^6^ Apollo Proton Cancer Centre, Chennai, India; ^7^ Department of General Surgery, Sir Run-Run Shaw Hospital, Zhejiang University, Hangzhou, China

**Keywords:** women in oncology, Hepato-pancreatic biliary cancer, HPB tumors, HCC, pancreatic cancer, cholangiocarcinoma

This editorial presents the inaugural Frontiers in Oncology ‘Women in HPB Tumors” series of article collections. The Research Topic collection highlights the diversity of research performed across the entire breadth of oncology research by women scientists pursuing STEM careers. Research articles published under this Research Topic aimed to present advances in theory, experiment, and methodology, with applications to compelling problems related to hepato pancreatic biliary cancers.

Exploring the tumor-immune interactions in the tumor microenvironment (TME) and identifying new prognostic and therapeutic biomarkers will assist in decoding the novel mechanism of tumor immunotherapy.

Clinical and *in vitro* work was done by authors contributing to articles published in the Research Topics using serum for ELISA, comet assay, qRT-PCR, IHC, Western, and genotyping, performed using allelic discrimination and confirmed by sequencing, or using various cancer cell lines followed by microscopy, assays for MTT, migration, colony and sphere formation, qRT-PCR, FACS, Western blot, tissue microarray, IHC and bioinformatics, and *in silico* analysis using the online databases.

The works explored by the research articles were based on *in-silico* and advanced bioinformatics analysis for evaluation of targets by The Cancer Genome Atlas (TCGA), which is a publicly available online database (https://www.cancer.gov/about-nci/organization/ccg/research/structural-genomics/tcga).

The freely available online database resource KEGG (Kyoto Encyclopedia of Genes and Genomes (https://www.genome.jp/kegg/) was used for the selection of relevant biological functions and pathways with enrichment scores of P<0.05, and the liver hepatocellular carcinoma (LIHC) dataset was downloaded from the Broad Institute TCGA Genome Data Analysis Center, https://doi.org/10.7908/C11G0KM9.

Also, STRING database (http://string-db.org) was utilized to construct PPI networks of coexpressed genes with interaction scores > 0.4. For visualization, CytoHubba, a plugin from the open-source platform Cytoscape (version 3.8.2) (http://www.cytoscape.org/), was employed to analyze and calculate the network structure.

Several concepts have been demonstrated or proved in the published articles (i) standard-of-care diagnostic biopsies and (ii) personalized therapy, and more.

HCC was addressed in 5 articles, while one presented colorectal cancer, however, pancreatic cancer was studied in 4 articles, by 89 authors.

The emerging importance of cancer personalized treatment plans is addressed in 3 research articles. As each human tumor creates its own unique microenvironment, He et al. claimed that assessment of BGN expression represents a promising approach for identifying patients with the greatest potential to benefit from immunotherapy and is a new venture into personalized therapy for colon cancer patients. BGN (a typical extracellular matrix (ECM) protein, validated as a signaling molecule regulating multiple processes of tumorigenesis), could serve as a valid biomarker for diagnosis, prognosis, and immunotherapy response prediction in patients with colon cancer.

Lundy et al.'s study demonstrates proof-of-principle feasibility to molecularly screen patients with pancreatic ductal adenocarcinoma for targeted therapies, and confirms diagnostic endoscopic ultrasound-guided fine-needle aspiration (EUS-FNA) biopsies. Single agent panitumumab was safe and tolerable but led to no objective tumor responses in the population tested.

In another study, Ren et al. revealed the clinicopathological features and identified risk factors of prognosis among patients with pancreatic cancer bone metastasis (PCBM), to be six independent predictors of prognosis, including age, pathological type, chemotherapy, liver metastasis, lung metastasis, and marital status. Knowledge of these survival predictors is helpful for clinicians to accelerate clinical decision process and design personalized treatment for patients with PCBM.

The crucial role of non-coding microRNAs (miRNAs) in pathogenesis of different diseases, including cachexia of strong pro-inflammatory environments, occurring in pancreatic and non-small cell lung cancer patients, was studied by Yehia et al. Where high levels of miR-155 in the cachectic group lead to negative feedback inhibition of both SOCS1 and the transcription factor Foxp3 in both the pancreatic and NSCL cancer patients.

Studies are also included on the roles of exosomes (Exo) in cancer development *via* mediating communication between tumor and its microenvironment. Hedgehog ligands undergo complicated post-translational modifications that result in lipid attachment and multimerization. Mutations in Hedgehog pathway components or induced Hedgehog signaling pathway components including Shh are found during injury or severe stress, or HBV or HCV infection and in HCC, as Li et al.'s group studied. They found higher plasma cancer cell-derived Exo-Shh levels associated with higher recurrence, suggesting Exo-Shh could serve as a prognostic biomarker and points to the possibility of tumor-secreted exosome being a therapeutic target.

As the demand for potential molecular biomarker(s) that can effectively predict prognosis and progression of HCC has increased, Ouyang et al. studied the anti-silencing function 1B (ASF1B) expression and function in HCC. They provided multi-level evidence for the significance of oncogenic gene *ASF1B* in HCC development and could be a target for inhibiting HCC cell growth, *via* inducing apoptosis and cell cycle arrest, reduced the expression of proliferating cell nuclear antigen (PCNA), cyclinB1, cyclinE2 and CDK9. These findings proposed a potential target for the development of anti-cancer strategies in HCC.

One of the major risk factors for HCC is hepatitis C virus (HCV) infection. The epithelial cell adhesion molecule (EpCAM) is a stem cell marker involved in the tumorigenesis and progression of many malignancies, including HCC, and was studied by Motawi et al.Serum EpCAM levels may hold promise for HCC diagnosis and for improving the diagnostic accuracy of α-fetoprotein.

Currently, there is a lack of tumor-selective and efficacious therapies for HCC. Zhao et al. studied the effect of the new chemotherapeutic agent β-Lapachone (β-lap; ARQ761 in clinical form) as a novel NADPH:quinone oxidoreductase 1 (NQO1) bioactivatable drug, selectively kills HCC cells expressing NQO1, through inducing ROS and PAR formation, NAD^+^ and ATP depletion and lethal DNA damage. High *NQO1:CAT* ratios in HCC tumors but low ratios in normal tissues offer an optimal therapeutic window and an ideal therapeutic target for β-lap.

Jin et al. studied the role of response to antiviral therapies on survival of patients with intermediate-stage HBV-related HCC undergoing transarterial chemoembolization (TACE). They proved the importance of regular HBV DNA surveillance and durable viral suppression during antiviral treatment.

Finally, Bai et al. studied the combination of the well-tolerated modalities tumor treating fields (TTFields) with mild hyperthermia of 38.5°C, as a novel supporting therapy combination for pancreatic cancer, more effective than each single treatment, with greater efficacy results without increased toxicity.

Great potential and efforts in cancer prognosis and therapeutics still need to be worked on. Therefore, we still need to do more and more research for efficient predictive and prognostic molecular-biomarkers. Moreover, we still need to address (epi)genetic profiling for precision medicine implementation, and (epi)genetic new novel potential targets identification and characterization for cancer treatment and/or control. Let us move ahead with drug design and discovery as well as drug repurposing for HPB tumors, with special emphasis on nano-bio-medicine.

## Publisher’s Note

All claims expressed in this article are solely those of the authors and do not necessarily represent those of their affiliated organizations, or those of the publisher, the editors and the reviewers. Any product that may be evaluated in this article, or claim that may be made by its manufacturer, is not guaranteed or endorsed by the publisher.

## Author Contributions

NMH was an associate editor of the Research Topic and wrote the paper text. YJ, SS, WW, YZ are guest associate editors for FIO and acted as editor for one paper in the Research Topic. BS was co-associate editor of the Research Topic. JC is a guest associate editor for FIO and acted as editor for two papers in the Research Topic. All authors contributed to the article and approved the submitted version.

## Conflict of Interest

The authors declare that the research was conducted in the absence of any commercial or financial relationships that could be construed as a potential conflict of interest.

## Publisher’s Note

All claims expressed in this article are solely those of the authors and do not necessarily represent those of their affiliated organizations, or those of the publisher, the editors and the reviewers. Any product that may be evaluated in this article, or claim that may be made by its manufacturer, is not guaranteed or endorsed by the publisher.

